# Potentially preventable hospital readmissions after patients’ first stroke in Taiwan

**DOI:** 10.1038/s41598-022-07791-3

**Published:** 2022-03-08

**Authors:** Ling-Jan Chiou, Hui-Chu Lang

**Affiliations:** 1 Department of Health Business Administration, Department of Nursing, and Department of Oral Hygiene, Meiho University, Pingtung County, Taiwan; 2 Institute of Hospital and Health Care Administration, National Yang Ming Chiao Tung University, 155, Sec.2, Li-Nong St., Taipei, 112 Taiwan

**Keywords:** Health care, Risk factors

## Abstract

Readmission is an important indicator of the quality of care. The purpose of this study was to explore the probabilities and predictors of 30-day and 1-year potentially preventable hospital readmission (PPR) after a patient’s first stroke. We used claims data from the National Health Insurance (NHI) from 2010 to 2018. Multinomial logistic regression was used to assess the predictors of 30-day and 1-year PPR. A total of 41,921 discharged stroke patients was identified. We found that hospital readmission rates were 15.48% within 30-days and 47.25% within 1-year. The PPR and non-PPR were 9.84% (4123) and 5.65% (2367) within 30-days, and 30.65% (12,849) and 16.60% (6959) within 1-year, respectively. The factors of older patients, type of stroke, shorter length of stay, higher Charlson Comorbidity Index (CCI), higher stroke severity index (SSI), regional hospital, public and private hospital, and hospital in the lower urbanized area were associated significantly with the 30-day PPR. In addition, the factors of male, hospitalization year, and monthly income were associated significantly with 1-year PPR. The ORs of long-term PPR showed a decreasing trend since implementing the national health insurance post-acute care (PAC) program in 2014 and a dramatic drop in 2018 after the government expanded the long-term care plan-LTC 2.0 in 2017. The results showed that better discharge planning, implementing post-acute care programs and long-term care plan-LTC 2.0 may benefit the care of stroke patients and help reduce long-term readmission in Taiwan.

## Introduction

As an important indicator of the quality of care, readmissions may occur because of events or conditions in the initial hospital stay, such as poor clinical care and poor coordination of services during hospitalization^1,2^, incomplete treatment of the underlying problem, and/or the development of a complication that becomes evident only after discharge^3^. Stroke patients have a high probability of being readmitted to the hospital after discharge. In most studies, 30-days post-discharge was identified as the most common readmission period^4^. The extant literature has provided the frequency of hospital readmission, ranging from 6.5 to 24.3% within 30-days^4–8^, and from 31 to 49% within 1-year^9–11^.

However, studies have demonstrated that some of these readmissions are unavoidable, even with optimal care^12^. Hospital readmission has also been highlighted as a source of significant healthcare expenditure and may constitute a potential target for cost savings, which supports the logic of using all-cause readmissions as a hospital performance metric^13^. Indeed, if readmission rates are considered a key indicator of hospital quality of care, readmissions can be identified as potentially preventable and/or unpreventable based upon clinical evidence criteria.

A readmission is defined as a subsequent hospitalization in an acute care hospital that follows a prior acute care admission within a specific time interval. Based upon administrative data, the potentially preventable readmission (PPR) method is used commonly to identify hospital readmissions that may indicate problems with a prior admission, and therefore be potentially preventable^14^. PPRs are those readmissions that could be avoided potentially given better clinical management, better stabilization of patients’ prior discharge, appropriate discharge planning, better outpatient treatment post-discharge, and resources at home that are sufficient to meet patients’ needs^15^. Overall, PPRs are events that could have been prevented with a better quality of hospital care, community care, and/or home care.

PPRs were defined according to AHRQ Prevention Quality Indicators (PQIs). The U.S. Agency for Healthcare Research and Quality (AHRQ) has used PQIs to define PPRs^16^. These PQIs constitute a set of evidence-based measures that use hospital inpatient administrative data to identify avoidable hospitalizations^8^. They have been employed widely to assess the quality of care and common ambulatory care-sensitive conditions^15,17–21^, many of which are related to readmission risk factors after stroke^9,15^.

Patient characteristics, social circumstances, health systems, clinical care processes, and health outcomes are potential factors in readmission after a stroke^5^. Predictors may vary and reflect different underlying mechanisms of the causes of readmission. Bjerkreim et al.^11^ found that the most frequent causes of readmission were infections, recurrent ischemic stroke, other cardiovascular events, and events associated with primary stroke. Patients readmitted early had a shorter length of index admission, poorer physical function, higher frequencies of an atherosclerotic etiology of index stroke, atrial fibrillation, and complications attributable to infection during index admission compared to patients readmitted late. Late readmission was correlated with older age and prior myocardial infarction. Readmissions within a short time post-discharge may indicate poor clinical care, unresolved problems at initial discharge, the quality of immediate post-hospital care, and a more chronically ill population^22^.

The strategies used to prevent short-term and long-term PPR should differ. It is important for organizations to identify the managerial strategies necessary to reduce patient readmission after stroke to improve the quality of care and save costs^4,23,24^. Nevertheless, few studies have targeted PPR after stroke using nationally-based data and few have compared the short- (30-day) and long-term (1-year) PPR after a patient’s first stroke. Thus, this study was designed to explore the preventable and non-preventable predictive factors that may influence readmission using nationwide population-based data.

## Materials and methods

### Data source

This study employed a population-based retrospective cohort study design. We used claims data derived from the National Health Insurance (NHI), Taiwan (LHID2005: Longitudinal Health Insurance Database 2005). This database consists of a national representative sample of one million individuals from the population of Taiwan overall. There were no statistically significant differences when age, sex, or insurance premium distributions of all NHI-enrolled individuals were compared^25^. The Taipei Veterans General Hospital Institutional Review Board reviewed and approved the study proposal (VGHIRB No. 2015-05-006BC#4). The database contained no identifiable personal information; hence waiver of informed content was granted. We confirm that all experiments were performed in accordance with relevant guidelines and regulations.

### Sample selection

**Figure 1:**
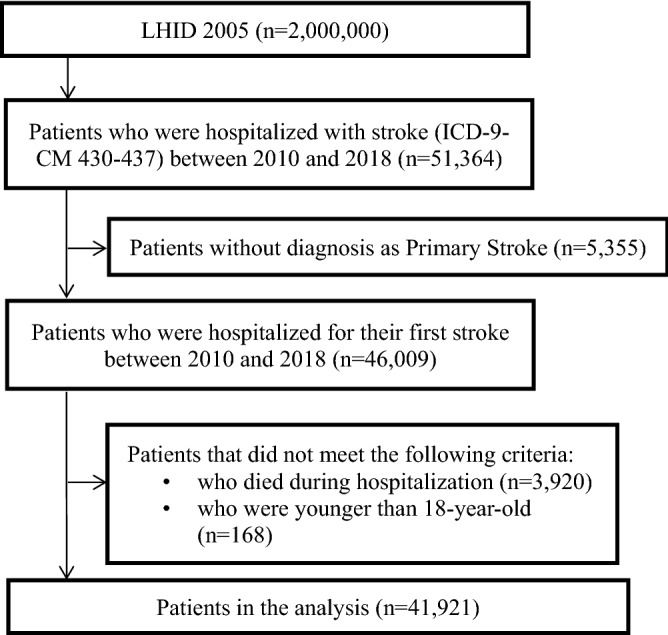
Flow chart of the data processing.

We included patients hospitalized for their first-ever stroke (ICD-9-CM 430-437) between 2010 and 2018 who were examined within 30 days with computed tomography (CT) or magnetic resonance imaging (MRI). We excluded patients with a stroke diagnosis before the index date, those who died during hospitalization, discharged themselves voluntarily, were transferred, had fewer than three outpatient visits within one year after discharge, had no insurance record, and were younger than 18-years-old. The final sample comprised 41,921 patients. The sample selection procedure is shown in Fig. 1.

### Causes of readmission

Preventable readmissions were defined according to the Agency for Healthcare Research and Quality (AHRQ) Prevention Quality Indicators (PQIs)^8,16^, which include chronic lung condition indicators (chronic obstructive pulmonary disease and adult asthma), diabetes-related preventable conditions (short- and long-term complications, and uncontrolled diabetes), cardiovascular-related indicators (hypertension, congestive heart failure, and angina without procedure), and acute condition indicators (dehydration, bacterial pneumonia, and urinary tract infection) (Supplementary Table S1). Non-PPR was defined as readmission after initial admission with a stroke diagnosis where the aforementioned diseases were not diagnosed at the time of readmission. Patients who were not readmitted to the hospital were defined as having no readmission.

### Covariates and subgroups

The covariates in this study included sex, age group, year of first admission for stroke, length of stay (LOS), stroke type (ISC: ischaemic stroke (codes 433–434), ICH: intracerebral hemorrhage (codes 431–432), SAH: subarachnoid hemorrhage (codes 430), and other (codes 435–437), monthly income based upon the NHI premium each patient paid, which was used as a proxy for income, hospital level, ownership, and region, and urbanization level, in which level 1 represents the most urbanized area and level 5 the least^26^. A modified version of the Charlson Comorbidity Index (CCI) was used to summarize comorbidities^27,28^. In the 1-year follow-up, the LOS included the initial hospitalization and hospital stays later during the year.

The comorbidities with PPR and non-PPR after discharge post-stroke were calculated according to 29 diagnosed diseases (Supplementary Table S2), which include any primary and secondary diagnosed conditions in outpatient or inpatient data during the period between the first admission for stroke and readmission post-discharge. The items included in the Stroke Severity Index (SSI) essentially reflect the management of stroke-related complications, and are generally correlated with stroke severity and other accompanying neurological deficits. We extracted the above claims information from the inpatient claims database at first admission for stroke and then computed each patient’s SSI. Following a previous study, patients were categorized as having mild (SSI ≤ 5), moderate (SSI 5 to ≤ 12), or severe (SSI > 12) stroke^29^.

### Data analysis

Descriptive statistics were used to summarize all of the covariates considered in this study, in which categorical variables were analyzed using Pearson’s Chi-squared test, and continuous variables (LOS) were analyzed using the *t*-test. In this study, separate models were built to examine the covariates associated with readmission status within 30-days and 1-year. Multinomial logistic regression (MLR) was performed to determine the association between related factors and readmissions. Three levels were defined for the dependent variable, readmission status: PPR patients, non-PPR patients, and no-readmission patients. Among them, no readmission was set as the reference level. The MLR results were presented as odds ratios (ORs), 95% confidence intervals (95% CIs), and *p*-values. Statistical significance was set at *p* < 0.05. All statistical analyses were performed with SAS software v. 9.4 (SAS Institute Inc., Cary, NC, U.S.A.).

### Ethics approval

Institutional Ethical Review (VGHIRB No. 2015-05-006BC#4).

### Consent for publication

All the authors have agreed this publication.

## Results

41,921 discharged stroke patients in total were identified during the study period. Table 1 shows the summary statistics of all covariates for the PPR, non-PPR, and non-readmission groups. Of these patients, 6490 (15.48%) were readmitted within 30-days, and 19,808 (47.25%) were readmitted within 1-year. Among them, the readmission rates for PPR and non-PPR were 4123 (9.84%) and 2367 (5.65%) within 30-days, and 12,849 (30.65%) and 6959 (16.60%) within 1-year, respectively.

**Table 1:** Factors associated with potentially preventable readmission (PPR), non- preventable readmission (non-PPR) and non-readmission patients within 30 days/1 year all after stroke (n = 41,921).

Variable	30 days							1 year						
	PPR		Non-PPR		Non-readmission		Chi-squared	PPR		Non-PPR		Non-readmission		Chi-squared
	n	%	n	%	n	%	*p-*value	n	%	n	%	n	%	*p-*value
N	4123	9.8	2367	5.7	35,431	84.5		12,849	30.7	6959	16.6	22,113	52.8	
**Sex**														
Male	2444	9.9	1447	5.9	20,790	84.2	0.054	7440	30.1	4223	17.1	13,018	52.8	< 0.001
Female	1679	9.7	920	5.3	14,641	84.9		5409	31.4	2736	15.9	9095	52.8	
**Age**														
18–44	184	7.3	225	8.9	2121	83.8	< 0.001	499	19.7	595	23.5	1436	56.8	< 0.001
45–64	1239	8.9	788	5.7	11,873	85.4		3449	24.8	2211	15.9	8240	59.3	
65–69	426	9.1	259	5.5	3994	85.4		1314	28.1	750	16.0	2615	55.9	
70–79	1071	10.1	550	5.2	9013	84.8		3469	32.6	1690	15.9	5475	51.5	
≧ 80	1203	11.8	545	5.4	8430	82.8		4118	40.5	1713	16.8	4347	42.7	
**Monthly income**														
≦ 19,047	1333	10.0	768	5.8	11,190	84.2	< 0.001	4219	31.7	2257	17.0	6815	51.3	< 0.001
19,048–21,900	1659	10.5	862	5.5	13,243	84.0		5111	32.4	2628	16.7	8025	50.9	
> 21,900	1131	8.8	737	5.7	10,998	85.5		3519	27.4	2074	16.1	7273	56.5	
**Year**														
2010	516	10.5	222	4.5	4196	85.0	< 0.001	1671	33.9	714	14.5	2549	51.7	< 0.001
2011	463	9.1	235	4.6	4397	86.3		1669	32.8	765	15.0	2661	52.2	
2012	503	10.4	235	4.9	4106	84.8		1665	34.4	702	14.5	2477	51.1	
2013	466	9.6	234	4.8	4132	85.5		1580	32.7	762	15.8	2490	51.5	
2014	466	9.9	255	5.4	3981	84.7		1491	31.7	769	16.4	2442	51.9	
2015	452	9.6	269	5.7	4001	84.7		1406	29.8	782	16.6	2534	53.7	
2016	426	9.9	289	6.7	3597	83.4		1261	29.2	888	20.6	2163	50.2	
2017	430	10.1	321	7.6	3501	82.3		1200	28.2	917	21.6	2135	50.2	
2018	401	9.5	307	7.3	3520	83.3		906	21.4	660	15.6	2662	63.0	
**Stroke type**														
ISC	2690	9.7	1476	5.3	23,635	85.0	< 0.001	8560	30.8	4378	15.8	14,863	53.5	< 0.001
ICH	885	13.1	497	7.4	5384	79.6		2348	34.7	1367	20.2	3051	45.1	
Other	477	7.2	332	5.0	5821	87.8		1733	26.1	1053	15.9	3844	58.0	
SAH	71	9.8	62	8.6	591	81.6		208	28.7	161	22.2	355	49.0	
LOS (mean, SD)	11.51	9.6	11.25	9.8	16.3	35.8	< 0.001	18.56	23.0	19.24	25.1	12.63	39.6	< 0.001
**CCI**														
0	1185	9.2	736	5.7	10,943	85.1	< 0.001	3216	25.0	1951	15.2	7697	59.8	< 0.001
1–3	2065	9.6	1119	5.2	18,390	85.2		6673	30.9	3353	15.5	11,548	53.5	
4–6	660	11.1	349	5.9	4949	83.1		2311	38.8	1191	20.0	2456	41.2	
≧ 7	213	14.0	163	10.7	1149	75.3		649	42.6	464	30.4	412	27.0	
**SSI**														
Mild	2855	8.4	1653	4.9	29,402	86.7	< 0.001	9304	27.4	5132	15.1	19,474	57.4	< 0.001
Moderate	364	13.4	198	7.3	2150	79.3		1002	37.0	503	18.6	1207	44.5	
Severe	904	17.1	516	9.7	3879	73.2		2543	48.0	1324	25.0	1432	27.0	
**Hospital level**														
Medical center	1368	9.1	863	5.8	12,757	85.1	< 0.001	4279	28.6	2672	17.8	8037	53.6	< 0.001
Regional hospital	1965	9.3	1102	5.2	18,014	85.5		6464	30.7	3302	15.7	11,315	53.7	
District hospital	789	13.5	402	6.9	4646	79.6		2103	36.0	983	16.8	2751	47.1	
**Hospital ownership**														
Public	1217	10.2	682	5.7	9984	84.0	< 0.001	3696	31.1	2019	17.0	6168	51.9	0.072
Private	898	10.9	493	6.0	6878	83.2		2589	31.3	1343	16.2	4337	52.5	
Legal person	2007	9.2	1192	5.5	18,557	85.3		6561	30.2	3595	16.5	11,600	53.3	
**Urbanization level**														
1	543	7.8	330	4.8	6075	87.4	< 0.001	1972	28.4	1114	16.0	3862	55.6	< 0.001
2	1211	10.5	746	6.5	9608	83.1		3437	29.7	1990	17.2	6138	53.1	
3	879	9.1	495	5.1	8290	85.8		2931	30.3	1571	16.3	5162	53.4	
4	705	10.0	398	5.7	5925	84.3		2234	31.8	1173	16.7	3621	51.5	
5	776	11.7	391	5.9	5453	82.4		2257	34.1	1093	16.5	3270	49.4	

The comparison results showed the significant covariates associated with readmission status within 30-days: age, monthly income, year, stroke type, LOS, CCI, SSI, hospital level, hospital ownership, and urbanization. For both 30-day and 1-year readmissions, the PPR rate of these significant factors was more likely to be higher than non-PPR, and the highest PPR rate of subgroups among the significant factors were one patient 80+, patients with a monthly income of NT$19,048–21,900, those treated in 2010 and 2012, those with ICH, those with CCI 7+, those with severe SSI, and those treated at district, private, and the least urbanized area hospitals.

The mean LOS within 30-days was 11.51 (SD = 9.59) for PPR and 11.25 (SD = 9.75) for non-PPR and within 1-year were 18.56 (SD = 22.97) for PPR and 19.24 (SD = 25.08) for non-PPR. The standard deviation of LOS within 1-year appeared to be large, which indicates that the LOS values were distributed over a broader range.

The results of the multinomial logistic regression are shown in Table 2. Compared to no readmission, patients 45–64, 65–69, 70–79, and 80+ years of age vs. those 18–44 years had ORs of 1.29, 1.33, 1.43, and 1.61 for 30-day PPR, and 1.20, 1.39, 1.66, and 2.37 for 1-year PPR. In addition, these ORs indicated that older patients have a higher probability of PPR. Compared to no readmission, within 30-days, LOS had ORs of 0.97 (95% CI [0.97–0.97]) and 0.97 (95% CI [0.97–0.97]) for PPR and non-PPR readmission, respectively. Within 1-year, LOS had an OR of 1.01 (95% CI [1.01–1.01]) and 1.01 (95% CI [1.01–1.01]) for PPR and non-PPR readmission, respectively. These ORs indicated that a shorter length of stay was associated with readmission within 30-days and had a greater effect on readmission within 1-year. Further, age played a key role in readmission.

**Table 2:** Factors associated with PPR and non-PPR *vs*. non-readmission after stroke (multinomial logistic regression).

Table	30 days				1 year			
	PPR vs. without readmission		Non-PPR vs. without readmission		PPR vs. without readmission		Non-PPR vs. without readmission	
	OR	95% CI	OR	95% CI	OR	95% CI	OR	95% CI
**Sex**								
Female								
Male	1.05	(0.98–1.12)	1.08	(0.99–1.18)	**1.07	(1.02–1.13)	***1.14	(1.08–1.21)
**Age**								
18–44								
45–64	**1.29	(1.09–1.52)	***0.68	(0.58–0.80)	**1.20	(1.07–1.34)	***0.66	(0.59–0.74)
65–69	**1.33	(1.10–1.60)	***0.66	(0.54–0.88)	***1.39	(1.22–1.58)	***0.67	(0.59–0.77)
70–79	***1.43	(1.20–1.69)	***0.62	(0.52–0.74)	***1.66	(1.48–1.86)	***0.71	(0.64–0.80)
≧ 80	***1.61	(1.35–1.91)	***0.62	(0.52–0.74)	***2.37	(2.11–2.67)	*0.88	(0.78–0.99)
**Monthly income**								
19,048–21,900								
≦ 19,047	1.07	(1.00–1.18)	1.02	(0.92–1.14)	***1.11	(1.04–1.17)	*1.08	(1.01–1.16)
> 21,900	1.07	(0.99–1.17)	0.99	(0.89–1.11)	*1.07	(1.01–1.13)	*1.09	(1.02–1.18)
**Year**								
2010								
2011	*0.84	(0.74–0.97)	1	(0.83–1.21)	*0.89	(0.81–0.98)	0.96	(0.85–1.08)
2012	0.99	(0.86–1.13)	1.07	(0.88–1.30)	0.97	(0.88–1.06)	0.95	(0.84–1.07)
2013	0.9	(0.78–1.03)	1.05	(0.86–1.27)	0.92	(0.84–1.00)	1.04	(0.92–1.17)
2014	0.95	(0.83–1.09)	1.19	(0.99–1.44)	*0.89	(0.81–0.98)	1.06	(0.94–1.19)
2015	0.95	(0.83–1.09)	**1.31	(1.09–1.57)	***0.81	(0.74–0.90)	1.06	(0.94–1.19)
2016	1.04	(0.90–1.19)	***1.66	(1.38–2.00)	**0.87	(0.79–0.96)	***1.44	(1.28–1.62)
2017	1.07	(0.93–1.23)	***1.88	(1.57–2.26)	***0.84	(0.76–0.93)	***1.52	(1.35–1.70)
2018	***0.99	(0.85–1.14)	***1.80	(1.50–2.16)	***0.49	(0.45–0.55)	***0.85	(0.75–0.96)
**Stroke type**								
Other type								
ISC	***1.44	(1.30–1.60)	1.06	(0.94–1.21)	***1.20	(1.12–1.28)	0.93	(0.86–1.01)
ICH	***1.97	(1.73–2.25)	**1.29	(1.10–1.52)	***1.40	(1.28–1.53)	1.08	(0.97–1.20)
SAH	**1.48	(1.13–1.95)	*1.39	(1.03–1.87)	1.17	(0.97–1.43)	1.17	(0.97–1.43)
LOS	***0.97	(0.97–0.97)	***0.97	(0.97–0.97)	***1.01	(1.01–1.01)	***1.01	(1.01–1.01)
**CCI**								
0								
1–3	1.06	(0.98–1.15)	1.01	(0.91–1.12)	***1.40	(1.33–1.48)	***1.28	(1.19–1.36)
4–6	***1.25	(1.12–1.39)	*1.18	(1.03–1.36)	***2.25	(2.09–2.42)	***2.15	(1.97–2.36)
≧ 7	***1.75	(1.48–2.06)	***2.41	(1.99–2.90)	***3.81	(3.33–4.36)	***4.94	(4.27–5.71)
**SSI**								
Mild								
Moderate	***1.84	(1.63–2.09)	***1.98	(1.68–2.32)	***1.60	(1.46–1.75)	***1.55	(1.38–1.73)
Severe	***3.69	(3.34–4.07)	***4.18	(3.69–4.74)	***3.27	(3.03–3.53)	***3.18	(2.90–3.47)
**Hospital level**								
District hospital								
Medical center	0.96	(0.88–1.04)	*0.90	(0.81–1.00)	**1.08	(1.02–1.14)	**0.92	(0.86–0.98)
Regional hospital	***1.37	(1.21–1.54)	*1.22	(1.04–1.42)	***1.40	(1.29–1.53)	*1.12	(1.00–1.24)
**Hospital ownership**								
Legal person								
Public	*1.11	(1.02–1.21)	1.02	(0.91–1.13)	1.03	(0.97–1.10)	1.04	(0.96–1.11)
Private	*1.12	(1.01–1.23)	0.99	(0.87–1.13)	1.03	(0.96–1.11)	0.98	(0.90–1.07)
**Urbanization level**								
1								
2	***1.38	(1.24–1.55)	***1.33	(1.15–1.53)	**1.13	(1.05–1.21)	*1.10	(1.01–1.21)
3	*1.17	(1.03–1.33)	0.98	(0.83–1.16)	*1.13	(1.04–1.24)	1.06	(0.95–1.18)
4	***1.28	(1.12–1.46)	1.18	(0.99–1.40)	**1.15	(1.05–1.26)	***1.15	(1.03–1.28)
5	***1.34	(1.16–1.55)	1.34	(1.16–1.55)	**1.24	(1.12–1.37)	1.21	(1.07–1.36)

*p < 0.05, **p < 0.01, ***p < 0.001.

**Figure 2:**
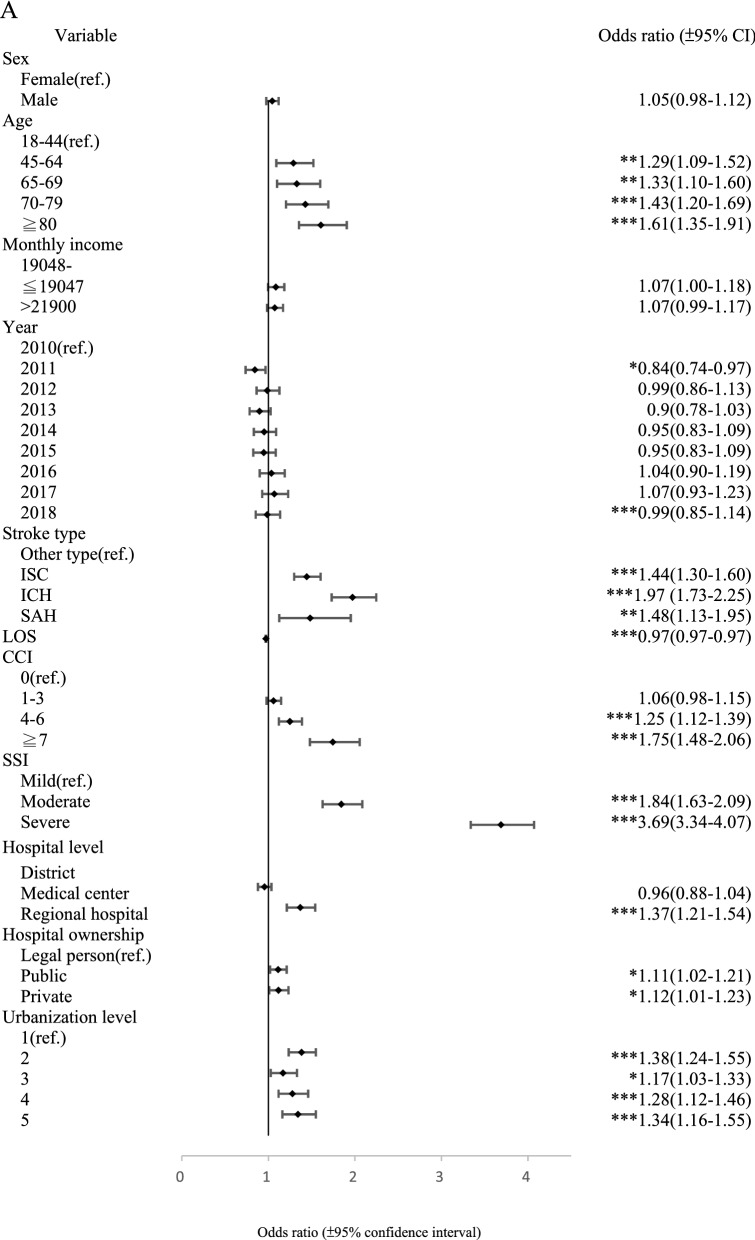

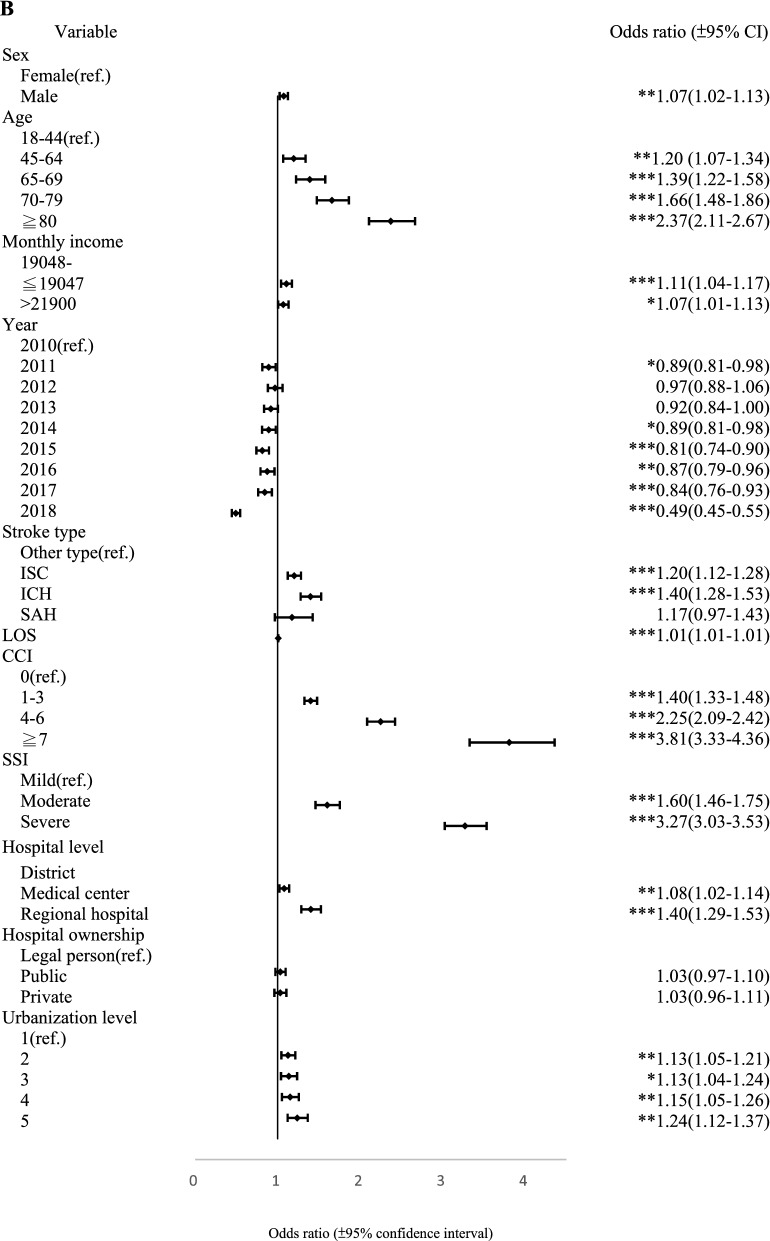
Forest plot displaying Odds ratios and 95% confidence intervals for 30-day (**A**) and 1-year PPR (**B**) after multinomial logistic regression. *p < 0.05, **p < 0.01, ***p < 0.001.

Within 30-days, the CCI level of 7+ and 4–6 (OR 1.75, 95% CI [1.48–2.06] and OR 1.25, 95% CI [1.12–1.39]), SSI levels of moderate and severe (OR 1.84, 95% CI [1.63–2.09] and OR 3.69, 95% CI [3.34–4.07]), treatment at a regional hospital (OR 1.37, 95% CI [1.21–1.54]) and hospital ownership, public and private (OR 1.11, 95% CI [1.02–1.21] and OR 1.12, 95% CI [1.01–1.23]), all types of stroke, all age levels and urbanization, were associated significantly with readmission. Similarly, all CCI levels (OR 1.40, 95% CI [1.33–1.48]; OR 2.25, 95% CI [2.09–2.42] and OR 3.81, 95% CI [3.33–4.36]), moderate and severe SSI levels (OR 1.60, 95% CI [1.46–1.75] and OR 3.27, 95% CI [3.03–3.53]), treatment at a medical center or regional hospital (OR 1.08, 95% CI [1.02–1.14] and OR 1.40, 95% CI [1.29–1.53]), and type of stroke, all age levels, and urbanization had a significant effect on readmission within 1-year. In addition to these covariates, male, hospitalization year, monthly income, and treatment at a hospital in the central area of Taiwan, also affected readmission significantly. Moreover, direct trends were discernable for age, CCI, SSI, and urbanization for PPR within 1-year, and age and SSI for PPR within 30-days. Further, Fig. 2 shows the forest plot of the odds ratios and 95% confidence intervals for factors associated with 30-day and 1-year PPR.

## Discussion

Studies have found that the rates of hospital readmissions after a stroke ranged from 6.5 to 24.3% within 30-days^5^ and 31 to 49% within 1-year^11^. However, not all readmissions are considered “potentially preventable”^30^. A review paper reported that preventable readmissions ranged from 14 to 23% within 30-days and from 48 to 59% within 1-year based upon older patients or general medical patients^15,31^. Another study estimated that the 1-year cumulative risks of readmission for ischemic stroke patients in Taiwan were 34.1%, 44.7%, and 62.9% for patients with mild, moderate, or severe stroke, respectively^32^. In this study, we determined that hospital readmission rates were 15.48% within 30-days and 47.25% within 1-year; the PPRs based upon the PQI definition were 9.84% within 30-days and 30.65% within 1-year using population-based data in Taiwan. Because our study included only patients older than 18, the readmission rates shown should be the upper bound.

Mittal et al. found that 41 (7.6%) of 537 acute ischemic stroke (AIS) patients were readmitted within 30-days post-stroke, and 2.8% among them were PPR^23^. Based upon 79 unplanned readmissions, an investigation at a Hong Kong geriatric center found that only 15 cases (19%) were avoidable^33^. These variations in PPR rates may be associated with age, patient diagnosis, duration of follow-up, methodology, and factors related to the mixtures of case diagnoses^15^. Nakagawa et al. found in a multiethnic population in Hawaii that 840 (8.4%) of 10,050 patients with any type of stroke-related hospitalization had 30-day PPR. Some studies have demonstrated that a higher readmission rate may be attributable to language barriers that affect receiving hospital care and/or accessing post-hospital care^30,34^.

The extant literature has reported that certain patient characteristics, such as age and socioeconomic status, were potential factors associated with readmission after stroke^5,8,15^. Our study found the same effect of age, but patients with the highest and lowest monthly income had a significantly higher rate of readmissions than those with the median income. This may indicate inequalities in healthcare and additional investigation is necessary to determine the reasons^35^. Regarding the age effect, the results showed that patients in the other age groups were at higher risk for short-term and long-term preventable readmissions than patients in the youngest age group. In contrast, the risk of non-preventable readmission was lower than in the youngest age group. It indicated that most readmissions in the younger age group are due to unpreventable factors.

The severity of stroke upon the first admission was also a significant predictor of 28-day readmission in Australia^5^. Further, CCI was found to be associated with the 30-day PPR after stroke discharge^30^. In this study, we identified a direct, positive relation between age, CCI, SSI, and long-term PPR. In cases of long-term PPR, the increase in these factors was associated with increasing readmission. These findings were similar to a 234 hospital-based study in Florida, which found that PPR was related to the severity of illness and older age. In addition, their results showed that increased severity of the disease and time between admission and readmission increased readmission rates^14^.

After adjusting for other variables, regional hospitals showed a higher risk of PPR compared to medical centers and district hospitals. The effect of hospital-level on short- and long-term readmissions was consistent with those of previous studies^32,36^. We assume that medical centers provide a better quality of inpatient care^32^, and suggest that regional hospitals’ policymakers give more attention to the quality of patient care. In addition, the fact that district hospitals had lower PPR than regional hospitals may be attributable to the implementation of the Post-Acute Care (PAC) program in Taiwan described in the next paragraph. The district hospitals received more PAC patients, which led to a decreasing readmission rate.

Our results showed that the hospitals’ urbanization level was related significantly to both short- and long-term PPR; the most urbanized area had the lowest readmission rate compared to the least urbanized area. One study suggested that this may be related to the poor quality of care in rural areas^37^. Most discharged stroke patients still need to receive follow-up healthcare at home or in a skilled nursing or inpatient rehabilitation facility; however, those resources may not be allocated sufficiently in rural areas compared to urban areas^38^. As a result, the quality of post-discharge care in rural areas may be poorer than that in urban areas and have led to a higher readmission rate.

Our study demonstrated further that, compared to no-readmission patients, a 1-day increase in LOS was associated significantly with 0.97 times the risk of 30-day PPR. However, a 1-day increase in LOS was associated significantly with 1.01 times the risk of 1-year PPR. Hence, LOS may have different implications for short- and long-term PPR. This finding is consistent with that in Bjerkreim’s study^11^. LOS’ short-term effect on PPR may be explained by incomplete treatment during the index hospitalization^14^, and suggests the need for a better quality of care and discharge planning. On the other hand, LOS’ long-term effect on PPR may be related to the severity of the stroke or comorbidities^10,39,40^, and suggests the need to improve the continuity of follow-up care.

The comorbidities associated with PPR diagnosed most frequently in our study were hypertension without complications, diabetes, and congestive heart failure. Previous reports have indicated that patients who were readmitted either early or later seemed to have higher frequencies of hypertension, atrial fibrillation, cerebrovascular disease, and diabetes as prior comorbidity conditions^10,11^. To decrease the risk of short-term PPR after discharge, our results showed that older patients, stroke type (ICH), CCI level of 4–6 and 7+, either moderate or severe SSI, and patients treated at regional, public or private, and hospitals in less urbanized areas are the groups most likely to experience a first-ever stroke, which suggest that adequate discharge planning must be provided for the first month after these patients are discharged. Although a previous study indicated that readmission reduction initiatives might not be highly effective for patients who are socioeconomically disadvantaged^41^, we found that more attention should be given to median-income patients to decrease readmission rates.

An important finding was that the ORs of long-term PPR vs. no readmission showed a decreasing trend. We believe that this is attributable to Taiwan’s implementation in 2014 of the national health insurance post-acute care (PAC) program for first-ever stroke patients. Patients who qualify for the PAC can receive intensive rehabilitation and integrated care within the treatment period. The PAC plan proposes to improve the incentives and review of discharge care for stroke patients in these hospitals. We suspect that the sudden drop in PPR and non-PPR readmission in 2018 might be due to the government expansion of the long-term care plan-LTC 2.0 in 2017, which encourages hospitals to provide more discharge plan services and offer more home-based medical care to the patients. The LTC-2.0 may benefit the care of stroke patients and decrease readmission. We suggest further research to verify this relationship.

PPR events may be avoided and healthcare costs reduced by improving the quality of care during the index inpatient stay and the period immediately following discharge. As a consequence, our study suggests that specific groups of patients should be targeted for PPR intervention.

## Limitations

This study has certain limitations. First, it was a retrospective cohort study with data derived from Taiwan’s National Health Insurance (NHI) claims database. Although we adopted rigorous definition of stroke to selection patients, overdiagnosis or underdiagnosis could not be overall excluded. In addition, data on certain important factors, such as patient behavioral characteristics, the process of care, and health-related quality of life could not be collected^23^. Nonetheless, compared to a hospital chart review database, the NHI claims database provides a population-based sample from a wide range of hospitals, as well as longitudinal follow-up information on readmission post-stroke. Second, the diagnosis codes’ accuracy was uncertain. Therefore, the results may be limited to patients who are hospitalized with a primary discharge diagnosis of stroke in Taiwan^36^. Third, identifying the risk of readmission may help with interventions to reduce readmissions. However, they cannot always be considered preventable, and readmission conditions such as the risk of deterioration of the chronic pulmonary disease, diabetes complications, heart failure, new stroke events, etc., cannot be eliminated entirely. Forth, Due to the data availability, we couldn’t include post-discharge death in our analysis. The results for the separate predictive model of death or potentially avoidable readmission may differ, and it is valuable for future researchers to explore it.

## Conclusion

We suggest that hospital managers provide better discharge planning and post-discharge follow-up programs for these patients before and after discharge, as the combination is likely to reduce the number of PPR substantially.

## Supplementary Information

Supplementary Tables.

## Data Availability

Data are sourced from Taiwan National Health Insurance (NHI). Due to legal restrictions imposed by the government of Taiwan in relation to the “Personal Information Protection Act”, data cannot be made publicly available. Requests for data can be sent as a formal proposal to the NHIRD (http://nhird.nhri.org.tw).
