# Supplementary material for: Potentially preventable hospital readmissions after patients’ first stroke in Taiwan
Source: Sci Rep. 2022 Mar 8;12:3743. doi: 10.1038/s41598-022-07791-3 (PMC8904540; doi:10.1038/s41598-022-07791-3)
Supplement: Supplementary file 1 — Supplementary Tables. [file 41598_2022_7791_MOESM1_ESM.docx]

**Supplementary Information**

Supplementary Table S1. Secondary diagnoses at the time of readmission linked to potentially preventable readmission (PPR)

| Diagnosis | ICD-9 |
| --- | --- |
| COPD | 490, 466.0, 491.0, 491.1, 491.20, 491.21, 491.8, 491.9, 492.0, 492.8, 494, 494.0, 494.1, 496 |
| Asthma | 493.0, 493.1, 493.2, 493.8, 493.9 |
| Diabetes | 250.1, 250.2, 250.3, 250.4, 250.5, 250.6, 250.7, 250.8, 250.9, 250.02, 250.03 |
| Primary hypertension | 401.0, 401.9, 402.00, 402.10, 402.90, 403.00, 403.10, 403.90, 404.00, 404.10, 404.90 |
| Heart failure | 398.91, 402.01, 402.11, 402.91, 404.01, 404.03, 404.11, 404.13, 404.91, 404.93, 428.0, 428.1, 428.2, 428.3, 428.4, 428.9 |
| Angina | 411.1, 411.81, 411.89, 413.0, 413.1, 413.9 |
| Volume depletion disorder | 481, 482.2, 482.30, 482.31, 482.32, 482.39, 482.41, 482.42, 483.0, 483.1, 483.8, 485, 486 |
| Pneumococcal pneumonia | 276.5 |
| Urinary tract infection | 590.0, 590.1, 590.2, 590.3, 590.8, 590.9, 595.0, 595.9, 599.0 |

Supplementary Table S2. Comorbidities associated with PPR, non-PPR, and non-readmission

|  | 30days | | | | | | | 1 year | | | | | | |
| --- | --- | --- | --- | --- | --- | --- | --- | --- | --- | --- | --- | --- | --- | --- |
|  | PPR | | non-PPR | | Without readmission | |  | PPR | | non-PPR | | Without readmission | |  |
|  | n | % | n | % | n | % | *p*-value | n | % | n | % | n | % | *p*-value |
| N | 4123 |  | 2367 |  | 35431 |  |  | 12849 |  | 6959 |  | 22113 |  |  |
| Hypertension Uncomplicated | 2340 | 56.75 | 956 | 40.39 | 18162 | 51.26 | <0.001 | 7577 | 58.97 | 2999 | 43.10 | 10882 | 49.21 | <0.001 |
| Diabetes | 1459 | 35.39 | 612 | 25.86 | 10836 | 30.58 | <0.001 | 4775 | 37.16 | 1933 | 27.78 | 6199 | 28.03 | <0.001 |
| Congestive Heart Failure | 1116 | 27.07 | 440 | 18.59 | 7865 | 22.20 | <0.001 | 3702 | 28.81 | 1415 | 20.33 | 4304 | 19.46 | <0.001 |
| Hypertension Complicated | 950 | 23.04 | 419 | 17.70 | 7093 | 20.02 | <0.001 | 3189 | 24.82 | 1311 | 18.84 | 3962 | 17.92 | <0.001 |
| Rheumatoid Arthritis/Collagen | 800 | 19.40 | 463 | 19.56 | 6713 | 18.95 | 0.617 | 2542 | 19.78 | 1443 | 20.74 | 3991 | 18.05 | <0.001 |
| Peptic Ulcer Disease excluding bleeding | 663 | 16.08 | 403 | 17.03 | 5167 | 14.58 | <0.001 | 2175 | 16.93 | 1231 | 17.69 | 2829 | 12.79 | <0.001 |
| Chronic Pulmonary Disease | 706 | 17.12 | 347 | 14.66 | 5076 | 14.33 | <0.001 | 2467 | 19.20 | 1061 | 15.25 | 2601 | 11.76 | <0.001 |
| Depression | 469 | 11.38 | 296 | 12.51 | 4275 | 12.07 | 0.330 | 1582 | 12.31 | 963 | 13.84 | 2495 | 11.28 | <0.001 |
| Cardiac Arrhythmia | 511 | 12.39 | 240 | 10.14 | 3612 | 10.19 | <0.001 | 1648 | 12.83 | 764 | 10.98 | 1951 | 8.82 | <0.001 |
| Liver Disease | 350 | 8.49 | 288 | 12.17 | 3245 | 9.16 | <0.001 | 1215 | 9.46 | 892 | 12.82 | 1776 | 8.03 | <0.001 |
| Fluid and Electrolyte Disorders | 319 | 7.74 | 148 | 6.25 | 1813 | 5.12 | <0.001 | 1011 | 7.87 | 479 | 6.88 | 790 | 3.57 | <0.001 |
| Renal Failure | 460 | 11.16 | 291 | 12.29 | 3356 | 9.47 | <0.001 | 1564 | 12.17 | 990 | 14.23 | 1553 | 7.02 | <0.001 |
| Other Neurological Disorders | 367 | 8.90 | 235 | 9.93 | 2883 | 8.14 | 0.003 | 1262 | 9.82 | 810 | 11.64 | 1413 | 6.39 | <0.001 |
| Valvular Disease | 236 | 5.72 | 102 | 4.31 | 1601 | 4.52 | 0.002 | 721 | 5.61 | 369 | 5.30 | 849 | 3.84 | <0.001 |
| Solid Tumor without Metastasis | 264 | 6.40 | 282 | 11.91 | 2314 | 6.53 | <0.001 | 923 | 7.18 | 840 | 12.07 | 1097 | 4.96 | <0.001 |
| Peripheral Vascular Disorders | 179 | 4.34 | 115 | 4.86 | 1419 | 4.00 | 0.087 | 585 | 4.55 | 326 | 4.68 | 802 | 3.63 | <0.001 |
| Hypothyroidism | 72 | 1.75 | 48 | 2.03 | 590 | 1.67 | 0.401 | 239 | 1.86 | 136 | 1.95 | 335 | 1.51 | 0.010 |
| Deficiency Anemia | 96 | 2.33 | 69 | 2.92 | 723 | 2.04 | 0.010 | 352 | 2.74 | 221 | 3.18 | 315 | 1.42 | <0.001 |
| Paralysis | 48 | 1.16 | 38 | 1.61 | 329 | 0.93 | 0.003 | 151 | 1.18 | 106 | 1.52 | 158 | 0.71 | <0.001 |
| Coagulopathy | 37 | 0.90 | 38 | 1.61 | 305 | 0.86 | 0.001 | 135 | 1.05 | 107 | 1.54 | 138 | 0.62 | <0.001 |
| Psychoses | 112 | 2.72 | 71 | 3.00 | 637 | 1.80 | <0.001 | 314 | 2.44 | 195 | 2.80 | 311 | 1.41 | <0.001 |
| Metastatic Cancer | 49 | 1.19 | 92 | 3.89 | 342 | 0.97 | <0.001 | 146 | 1.14 | 224 | 3.22 | 113 | 0.51 | <0.001 |
| Drug Abuse | 47 | 1.14 | 45 | 1.90 | 446 | 1.26 | 0.019 | 149 | 1.16 | 128 | 1.84 | 261 | 1.18 | <0.001 |
| Pulmonary Circulation Disorders | 22 | 0.53 | 7 | 0.30 | 116 | 0.33 | 0.094 | 57 | 0.44 | 37 | 0.53 | 51 | 0.23 | <0.001 |
| Obesity | 10 | 0.24 | 4 | 0.17 | 98 | 0.28 | 0.586 | 36 | 0.28 | 12 | 0.17 | 64 | 0.29 | 0.242 |
| Alcohol Abuse | 25 | 0.61 | 24 | 1.01 | 222 | 0.63 | 0.071 | 87 | 0.68 | 74 | 1.06 | 110 | 0.50 | <0.001 |
| Lymphoma | 18 | 0.44 | 18 | 0.76 | 94 | 0.27 | <0.001 | 41 | 0.32 | 37 | 0.53 | 52 | 0.24 | <0.001 |
| Weight Loss | 9 | 0.22 | 9 | 0.38 | 79 | 0.22 | 0.300 | 41 | 0.32 | 26 | 0.37 | 30 | 0.14 | <0.001 |
| AIDS/HIV |  |  |  |  |  |  |  | 5 | 0.04 | 8 | 0.11 | 13 | 0.06 | 0.117 |
